# Understanding knowledge, attitude and practices of voluntary blood donations among medical students to strengthen safe transfusion practices

**DOI:** 10.6026/973206300212054

**Published:** 2025-07-31

**Authors:** Nikita Singh Gaharwar, Vikas Pandey, Surabhi Mishra, Arjun Singh

**Affiliations:** 1 Department of Pathology, Government Medical College, Satna, Madhya Pradesh, India; 2 Department of Pathology, Government Medical College, Datia, Madhya Pradesh, India

**Keywords:** Blood donation, medical students, voluntary, knowledge, attitude

## Abstract

Knowledge, attitudes and practices regarding blood donation among 478 medical students in Central India is of interest. Only 46.2% had good overall knowledge and key eligibility criteria were known by just over half. Despite limited knowledge, 72.6% held a positive attitude toward voluntary donation. Fear of pain was the major barrier to donation. Thus, blood donation education into the medical curriculum is important.

## Background:

There are no alternatives to human blood, which is a necessary component of life [1]. Improving medical treatment requires having access to safe blood and blood products. To fulfill the majority of a nation's blood needs, WHO estimates that 1% of the population must donate blood [2]. In places like India, there are only around 2.5 donations for every 1000 eligible people, which is far less than the amount of blood that is needed [3]. Blood donors come in three varieties: replacement, paid professional and volunteer non-remunerated donors [4]. About 45% of blood donations in India are made by replacement donors, often known as family donors, who provide blood to friends, family, or other family members as necessary [5]. The WHO emphasizes that voluntary, non-paid blood donation must take the place of replacement blood donation, which should be discouraged. According to the Honorable Supreme Court's ruling, the nation outlawed the professional donor system on January 1, 1998 [6]. One crucial step in ensuring the safety, quality, accessibility and availability of blood transfusions is the collection of blood only from willing, unpaid donors from low-risk groups [7]. In order to get 100% regular voluntary blood donation, the WHO also urges nations to concentrate on youth [8].

An individual's mind-set, beliefs, and familiarity with blood donation significantly affect their willingness to donate. Research indicates that factors influencing these outcomes included knowledge, previous residence, and college background. Reluctance to donate blood was primarily linked to fear and concerns about time constraint [9]. Young students make up a larger share of the Indian population and are healthy, energetic, dynamic, resourceful and responsive. An individual's mindset, beliefs, and familiarity with blood donation significantly affect their willingness to donate. Research indicates that factors influencing these outcomes included knowledge, previous residence, and college background. Reluctance to donate blood was primarily linked to fear and concerns about time constraint. It is necessary to inspire, motivate and urge these young students to regularly give blood willingly. Therefore, it is of interest to understand the knowledge, attitudes and practices of voluntary Blood donors among medical students in Central India.

## Materials and Methods:

A standardized questionnaire was developed to evaluate the knowledge, attitudes and habits about blood donation among medical students. The assessment included socio-demographic characteristics, donors' understanding of blood donation eligibility requirements, their attitudes and intentions about first-time donations, their desire to contribute consistently to voluntary sources and the effects of blood donation. All research facts and parameters were elucidated to the participants and the questionnaire was administered subsequent to obtaining their agreement.

## Evaluation of the outcome variables:

## Knowledge:

Ten questions were employed to evaluate the existing knowledge of medical students on blood donation, scored as 0 or 1 for each correct response, yielding a total score between 0 and 10. The knowledge scores were classified as bad (49.9% or lower), fair (50% to 74.9%), or good (above 75%).

## The attitude:

Five questions were employed to evaluate the participants' opinions about voluntary non-remunerated blood donation with a 5-point Likert scale: strongly disagree (1), disagree (2), neutral (3), agree (4) and highly agree (5). The minimum attitude score was 5, while the highest score was 25. The ratings were transformed into percentages and evaluated as negative or positive attitudes. Assessment of participants' practice of voluntary blood donation in the past and their prospective intentions to contribute, together with reasons for abstaining from donation, was conducted using a limited number of questions. The findings are displayed as proportions.

## Data analysis:

The obtained data were rectified for response discrepancies, coded and analyzed utilizing the IBM Statistical Package for Social Sciences (SPSS version 20). The statistics are represented as percentages or averages and displayed in tables and charts.

## Results:

A total of 478 medical students, aged 18 to 27 years, participated in this study. Among the total participants, 55.86% (267/478) were male, while 44.14% (211/478) were female. Only 54.4% of students were cognizant of the minimum age required to begin blood donation, although 53.3% understood the suitable time between two donations. 62.1% of the pupils met the minimal weight requirement, while 57.5% satisfied the hemoglobin level criteria for blood donation. The awareness regarding the volume of blood provided in a single donation, the blood components derived from a single unit and the compulsory tests conducted on donated blood was 56.9%, 47.3% and 41.2%, respectively (Table 1 - see PDF). In all, 46.2% of students possessed proficient knowledge on voluntary blood donation, 28.1% exhibited moderate understanding and 25.7% showed inadequate knowledge (Figure 1 - see PDF). 72.6% of students advocated for the encouragement of voluntary donations, while 67.2% asserted that blood donation positively impacts health. 35.7% of the students believed that blood donation may result in complications such as weakness, anemia and infections. Over fifty percent of the students (53.7%) contended that blood donations should be restricted to friends and family, whilst 39.6% asserted that only men should be eligible to donate blood. A mere 14.2% of students believed that monetary reward should be offered for blood donation (Table 2 - see PDF). Seventy-five point three percent of the students had never donated blood, but eighty-three point nine percent expressed their willingness to donate blood regularly. The predominant reasons for abstaining from blood donation included anxiety and ambiguity over the donation process (74.2%), lack of opportunity (67.5%), ignorance of donation locations (62.3%) and apprehension about potential health problems such as weakness, anemia and weight loss post-donation (42.1%). A minor percentage of students (11.5%) anticipated some form of recompense (monetary or otherwise) for blood donation (Table 3 - see PDF).

## Discussion:

The participation of youth in voluntary blood donation is essential for fulfilling the need for safe blood and an increasing number of medical students possess a deeper comprehension of the healthcare requirements of our nation. Consequently, comprehending the diverse factors influencing the knowledge, attitudes and practices of voluntary blood donation among medical students is essential. The current study revealed that 74.4% of students were aware of the minimum age for initiating blood donation; these results align with another research conducted by Uma *et al.* [10] (79.4%) and Ugwu *et al.* [11] (86.7%). In the current study, 63.4% of students demonstrated awareness of the appropriate interval and frequency for blood donations, in contrast to similar studies conducted by Sahoo *et al.* [12], which reported awareness rates of 48.9%. In the current study, 47.3% of students were aware of the components derived from one unit of whole blood, compared to 63.9% in the research conducted by Devi *et al.* [13] and 64% in the study by Aslami *et al.* [14]. The awareness of component preparation was significantly lower in the study conducted by Chauhan *et al.* [15] (22%). The survey indicated that around 46.2% of the students have a strong understanding of voluntary blood donation. The results align with those of Ogboghodo *et al.* [16] and Karakkamandapam *et al.* [17], who indicated a strong understanding of voluntary blood donation among medical students. Aslami *et al.* [14] showed a minimal number of students possessing enough knowledge, likely due to their study's focus just on first and second-year medical students, which may account for the diminished understanding of blood donation. The study participants, who were second professionals or above, had previously, attended lectures in hematology and blood transfusion medicine; hence, their exposure to these lectures may have enhanced their understanding overall. The overall disposition of students about blood donation was favorable, with the majority concurring that it is a commendable practice that warrants encouragement, aligning with the findings of Meinia *et al.* [18] (94.6%), Salem *et al.* (96.6%) [19] and Nwogoh *et al.* (89.3%) [20]. Notwithstanding adequate awareness of blood donation, 53.7% of the students contended that blood should exclusively be donated to friends and relatives in times of necessity; a similar survey by Sabu *et al.* indicated that 15.2% of students held this belief, while 39.6% said that only men should engage in blood donation. In the current survey, 11.3% of the students said that some form of financial reward should be offered to donors in return for voluntary blood donation. Karakkamandapam *et al.* [17] showed that 15.2% of students anticipated financial remuneration for blood donation.

In the current survey, 75.3% of students acknowledged that they had never donated blood, despite a significant proportion possessing enough information and awareness regarding blood donation. Comparable outcomes were noted by Nwogoh *et al.* (77%) [20]. In contrast, Meinia *et al.* [18] indicated that 43.4% of the students participating in their research had previously donated blood. Notably, 83.9% of the students in this research agreed to give blood frequently, aligning with the findings of Sahoo *et al.* [12] (75.54%). The primary reasons for the student population's reluctance to donated blood included apprehension over the procedure or discomfort, insufficient opportunities and understanding about blood donation and concern about contracting illnesses through the process. Consequently, the current study, along with other analogous research, indicates that knowledge and awareness about blood donation are ordinary or subpar in over half of the student demographic, which is concerning considering their affiliation with the healthcare profession. Furthermore, although medical students possess some awareness of the necessity for blood donation, the practice remains significantly inadequate among them. Promoting blood donation among students at a young age and educating them about blood and the necessity of voluntary donation early in their healthcare journey prepares them to inform the public, engage in community health initiatives and make informed decisions in patient care, thereby enhancing overall healthcare outcomes.

## Conclusion:

A section on blood banking and transfusion medicine, along with regular awareness drives and donation camps should be included in the current undergraduate medical curriculum. This not only in stills a sense of social responsibility among the students but also helps in creating positive attitudes and removing misconceptions about donation. This knowledge and positive attitude will be beneficial for making informed decisions regarding patient healthcare.
